# Midbrain lesion-induced disconjugate gaze: a unifying circuit mechanism of ocular alignment?

**DOI:** 10.1007/s00415-023-12155-6

**Published:** 2024-02-14

**Authors:** Maximilian U. Friedrich, Laurin Schappe, Sashank Prasad, Helen Friedrich, Michael D. Fox, Andreas Zwergal, David S. Zee, Klaus Faßbender, Klaus-Ulrich Dillmann

**Affiliations:** 1 Center for Brain Circuit Therapeutics, Brigham and Women’s Hospital, 60 Fenwood Rd, Boston, MA 02115 USA; 2 Harvard Medical School, Boston, USA; 3 Department of Neurology, Saarland University Medical Center, Homburg, Germany; 4 Department of Neurology, University of Pennsylvania Perelman School of Medicine, Pennsylvania, USA; 5 German Center for Vertigo and Dizziness, University Hospital, LMU Munich, Munich, Germany; 6 Department of Neurology, University Hospital, LMU Munich, Munich, Germany; 7 Departments of Neurology, Ophthalmology, Otolaryngology, Head and Neck Surgery, The Johns Hopkins University School of Medicine, Baltimore, USA

**Keywords:** Eye movement disorders, Visual system, Vestibular system, Videooculography, Neuroimaging, Posterior commissure

## Abstract

**Background:**

Disconjugate eye movements are essential for depth perception in frontal-eyed species, but their underlying neural substrates are largely unknown. Lesions in the midbrain can cause disconjugate eye movements. While vertically disconjugate eye movements have been linked to defective visuo-vestibular integration, the pathophysiology and neuroanatomy of horizontally disconjugate eye movements remains elusive.

**Methods:**

A patient with a solitary focal midbrain lesion was examined using detailed clinical ocular motor assessments, binocular videooculography and diffusion-weighted MRI, which was co-registered to a high-resolution cytoarchitectonic MR-atlas.

**Results:**

The patient exhibited both vertically and horizontally disconjugate eye alignment and nystagmus. Binocular videooculography showed a strong correlation of vertical and horizontal oscillations during fixation but not in darkness. Oscillation intensities and waveforms were modulated by fixation, illumination, and gaze position, suggesting shared visual- and vestibular-related mechanisms. The lesion was mapped to a functionally ill-defined area of the dorsal midbrain, adjacent to the posterior commissure and sparing nuclei with known roles in vertical gaze control.

**Conclusion:**

A circumscribed region in the dorsal midbrain appears to be a key node for disconjugate eye movements in both vertical and horizontal planes. Lesioning this area produces a unique ocular motor syndrome mirroring hallmarks of developmental strabismus and nystagmus. Further circuit-level studies could offer pivotal insights into shared pathomechanisms of acquired and developmental disorders affecting eye alignment.

**Supplementary Information:**

The online version contains supplementary material available at 10.1007/s00415-023-12155-6.

## Introduction

Lesions or stimulation within the dorsal midbrain induce unique ocular motor patterns [1–3]. This region is neuroanatomically complex, owing to its role as a nexus for numerous interconnecting pathways and nuclei, integral to controlling eye movements [4]. Dorsal midbrain lesions typically impair conjugate vertical gaze but can also induce disconjugate eye movement disorders. These encompass *tonic* deficits such as ocular misalignment in the vertical and horizontal plane [5, 6] as well as *dynamic* deficits, such as disconjugate oscillations like hemi-seesaw nystagmus (HSSN) [7, 8] or convergence(-retraction) nystagmus (CRN) [5].

HSSN features vertical eye movements in *opposite* directions and torsional movements in the *same* direction. HSSN usually overlays a tonic vertical ocular misalignment: the ocular tilt reaction (OTR), consisting of head tilt, eye counter-roll, and skew deviation [1]. The vestibular graviceptive system, which integrates signals from otolith organs and semicircular canals, is thought to underlie both tonic and dynamic components [4, 8]. Its closely related, symmetrical counterpart, seesaw nystagmus (SSN), is associated with congenital or acquired abnormalities in the visual pathway and infantile strabismus [9]. Fixation and eye position commonly influence (hemi-)seesaw nystagmus, hinting at visual and vestibular contributions [9, 10].

CRN, in contrast, involves eye movements along the depth plane, oscillating between convergence and divergence [4]. A typical finding in Parinaud’s or dorsal midbrain syndrome [4], CRN overlays a tonic horizontal ocular misalignment, most often esodeviation [5, 6]. Some occurrences of CRN feature a globe retraction during the convergence phase. Despite over a century of keen scientific interest, the neuroanatomy and exact physiology of CRN remains elusive, likely owing to heterogeneous patterns observed in oculographic and neuroanatomic studies [5, 11–13].

We here characterize a patient who acutely developed disconjugate eye movements in both the vertical and horizontal plane, encompassing both HSSN and CRN, as a result of a solitary, focal midbrain ischemia. We used repeated binocular videooculography (VOG) to characterize eye movements and track their development. Concurrently, we combined advanced neuroimaging and neuroanatomical atlases to determine lesion topography.

## Methods and results

### Clinical assessment

**Fig. 1:**
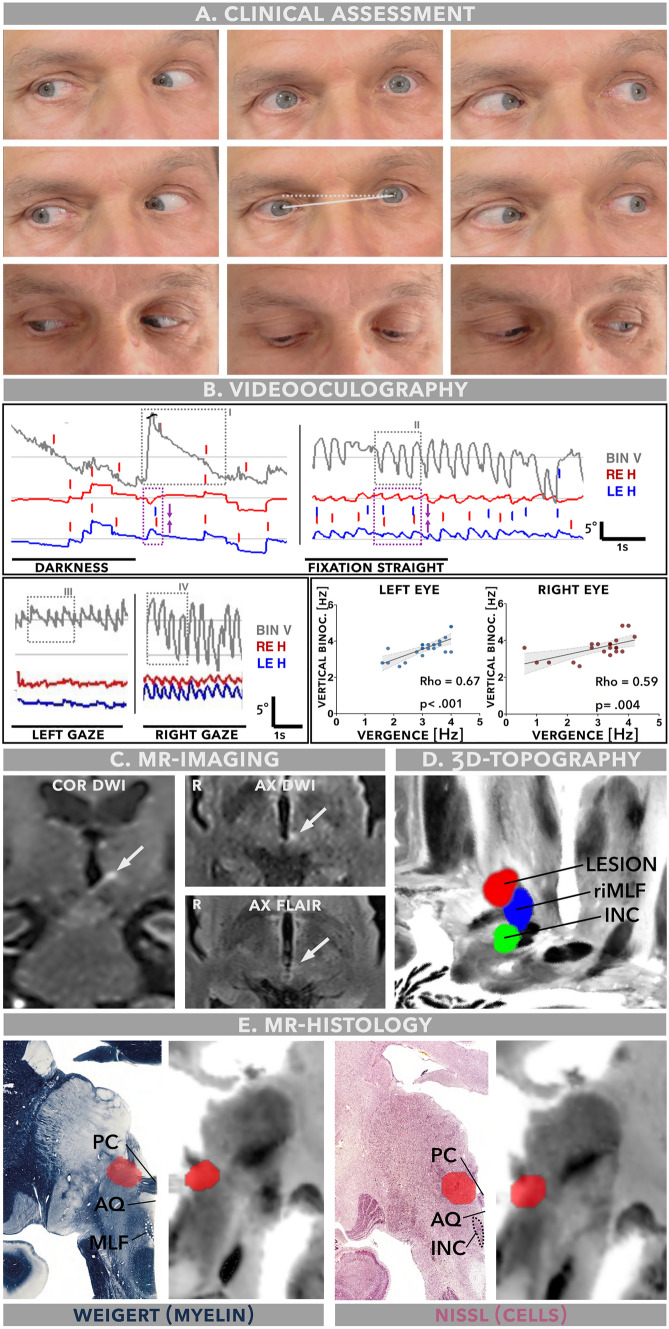
Summary of clinical findings, videooculography and neuroimaging. **A** Clinical neuroophthalmologic assessment shows a left hypertropia, rightward head deviation in the roll plane (ear to shoulder; dashed line approximation of a true horizontal, solid line approximation of patient’s actual head deviation) and esodeviation with right eye deviating inwards, greater on attempted upgaze. Vertical but not horizontal gaze range is limited. **B** Binocular videooculography. In darkness, the binocular vertical trace (light grey) shows a low frequency nystagmus with an upward directed quick phase and linear slow phase waveforms (highlighted in grey box I), co-occurring with a sparse convergent jerk (purple box) with following slow divergence visible in the horizontal trace. With fixation in light, the vertical and horizontal oscillations become significantly more intense and assume pendular waveforms (highlighted in grey box II and purple box). In left gaze, the horizontal oscillations are markedly attenuated, while vertical oscillations show a gaze-dependent shift of waveforms (highlighted in grey box III and IV). Vertical and horizontal oscillations are strongly correlated in both eyes. BIN V = binocularly averaged vertical trace, LE H = left eye horizontal, RE H = right eye horizontal. **C** MR-imaging (4 mm isotropic) acquired in the acute setting reveals a solitary, 3.8 × 4.2 mm ischemic lesion (white arrows) at the meso-diencephalic junction. COR = coronal, AX = axial, R = right. **D** The lesion (red) abutted the rostral interstitial nucleus of the medial longitudinal fasciculus (riMLF, blue) but not the interstitial nucleus of Cajal (INC, green). **E** Coronal reconstructions of the lesion (red), co-registered with a high-resolution (400 µm) MR-histology atlas (Juelich BigBrain, right half of image) and juxtaposed with matching histomorphological sections stained for myelin (Weigert, sudan black stain) and nuclei (kresyl violet, “Nissl”, both left side of image) reproduced with permission from (Mai JK, Majtanik M, Paxinos G. Atlas of the Human Brain. Academic Press; 2015). The lesion abuts the posterior commissure (PC) as well as an adjacent, Nissl-intense area of the dorsal midbrain, but not the interstitial nucleus of Cajal (INC) or the medial longitudinal fasciculus (MLF). Arrows also point to the aqueduct (AQ) for orientation

A 63-year-old man emergently referred himself after waking up with double vision and a sense that the environment was moving, an unsteady gait, dizziness, and a mild headache. He had no history of visual or ocular motor dysfunction and strabismus and reported normal vision in both eyes. Neuroophthalmological assessment (Fig. 1A) showed a rightward head tilt and a mild skew deviation with left eye hypertropia upon cover testing, consistent with a rightward ocular tilt reaction. There was a tonic horizontal misalignment: esodeviation with right eye turning inwards, greater in distance and upgaze, but without associated abduction deficit, consistent with non-paretic esotropia. Limited upgaze could be partly overcome by vertical vestibular-ocular reflex testing, suggesting a supranuclear etiology.

There was a multivectorial, disconjugate nystagmus consisting of dissociated vertical (i. e. one eye up, the other down) and conjugate torsional elements (*i. e.* the top pole beating towards the right ear), consistent with HSSN. In the horizontal plane, irregular convergent–divergent oscillations were accompanied by globe retraction, most prominent with upgaze effort, typical for CRN. The convergent movements were accompanied by a remarkable bilateral excyclotorsion (i. e. upper eye poles rotating towards the ipsilateral ear). Right gaze intensified convergent–divergent and vertical components, while in left gaze, conjugate torsional components were more pronounced. Fixation suppression with illuminated Frenzel goggles markedly reduced vertical oscillations while CRN remained unchanged (Supplementary Video).

There were no other neurological abnormalities such as light-near-dissociation. Over the course of acute clinical management (5–7 h) including emergency MR-imaging, the initial HSSN pattern evolved into a predominantly upbeating pattern more pronounced in the right eye.

### Videooculography

Binocular videooculography ~ 7h after admission confirmed limitation of vertical gaze range and saccades to ~ 5° from midline (Supplementary Fig. 1A).

In darkness, the horizontal channel showed a left beating spontaneous nystagmus with linear slow phases of 1–2.5°/s, superimposed on disconjugate, convergent drifts, more pronounced in the right eye, with a median frequency of 0.2Hz (Fig. 1B, purple box). The binocularly averaged vertical channel showed an upbeat nystagmus with linear slow phases and a median frequency of 0.7Hz (Fig. 1B, grey box I). Vertical and horizontal oscillation frequencies were uncorrelated in darkness.

During fixation in light, the horizontal channel showed convergent–divergent oscillations with mostly pendular waveforms and significantly higher frequency (3.7Hz vs. 0.2Hz, *P* = 0.03, Fig. 1B, purple box). The vertical oscillations shifted to pendular waveforms and showed a similar increase of oscillation frequency from 0.7 to 3.7Hz (*P* = 0.03, Fig. 1B, grey box II).

Gaze position affected vertical and horizontal oscillation frequency (gaze direction x nystagmus plane interaction, F(2) = 3.98, *P* = 0.004) and waveform (Fig. 1B, grey box III& IV). Left gaze position reduced horizontal and vertical oscillation frequency to 2.4Hz (*P* = 0.002) and 3.2Hz (*P* = 0.016), respectively. During fixation in light, vertical and horizontal oscillation frequencies were strongly correlated (left eye: rho = 0.67, *P* < 0.001, right eye: rho = 0.59, *P* = 0.0041, Fig. 1B).

Three days later, vertical gaze and saccade range was normalized and no ocular oscillations were detectable (Supplementary Fig. 1C).

### Lesion topography

Diffusion-weighted MRI revealed a solitary, acute ischemic lesion (~ 3.8 × 4.2mm) in the left meso-diencephalic junction (Fig. 1C). Topographical analysis revealed a lesion localization directly adjacent to the posterior commissure (PC), abutting the vertical saccade generator, rostral interstitial nucleus of the medial longitudinal fasciculus (riMLF)^4^ but not the vertical gaze integrator, interstitial nucleus of Cajal (INC) [1, 4, 7] (Fig. 1D–E).

## Discussion

### The functional neuroanatomy of disconjugate eye movements

In frontal-eyed animals, disconjugate eye movements serve a primary teleological purpose: precisely aligning objects in 3D space onto each eye’s fovea, producing the needed vertical and horizontal vergence [4] and thereby enabling depth perception. Disconjugate eye movements also support fixation and reduction of motion parallax during head movements in 3D space, via the translational vestibulo-ocular reflex (TVOR) [14].

Distinct midbrain regions have been associated with disconjugate horizontal [4–6] *or* vertical [1, 2, 7, 8] eye movements. The dorsal midbrain has been implicated *both* in hemi-seesaw and convergence–retraction nystagmus [5, 6, 8]. Simultaneous occurrence however is rare and has only been reported with extensive subcortical lesions [8], limiting precise neuroanatomical conclusions. Here, MRI revealed a solitary focal midbrain lesion adjacent to the posterior commissure, hinting at a circumscribed neuroanatomical origin for both oscillations. The lesion abuts the riMLF, consistent with the vertical saccade deficit [4], but not the INC. The area surrounding the INC, but not the nucleus itself, is critical for disconjugate vertical nystagmus [7, 8]. Experimental lesions directly lateral to the posterior commissure cause disconjugate, 2–4 Hz seesaw and convergence–divergence oscillations which are modulated by vestibular and visual inputs [3, 7]. These findings closely correspond to our patient’s lesion location and mirror the observed disconjugate 3.7Hz oscillations. Moreover, vertically acting eye muscles relax during convergence, presumably via inhibitory INC projections crossing through the PC [4, 15]. Thus, this pathway’s lesion-induced disconnection may induce globe retraction via inappropriate eye muscle co-contraction [4, 5, 13].

### Visuo-vestibular interactions and disconjugate eye movements

Ocular and postural control depends on the fusion of visual and vestibular signals into a congruent percept of self in space [4, 16]. Defective visuo-vestibular integration results in fixation-dependent oscillations like (hemi-)seesaw and infantile nystagmus [8, 9], but has not yet been linked to CRN. Our data suggest a close relationship of HSSN and CRN and therefore suggest a common substrate for vertically and horizontally disconjugate eye movements. The shared effects of fixation and illumination imply retinoreceptive neural circuits of the midbrain pretectum and accessory optic system (AOS) [16].

The AOS is a primordial visual system which has been implicated in developmental strabismus and nystagmus [9–11, 16]. Emerging theories propose a resurgence of AOS-related ocular motor patterns, which link disconjugate eye movements to atavistic balance reflexes in lateral-eyed species [9, 10, 16]. These encompass stereotypical patterns of esodeviation, vertical divergence and disconjugate fixation nystagmus [9, 10, 16], closely mimicked by this case. Additionally, binocular excyclotorsion co-occuring with convergent oscillations suggests inappropriate inferior oblique activation, yet another AOS-related phenomenon in developmental strabismus [16].

Densely interconnected via the posterior commissure, the pretectum and AOS also receives visual-related cortical inputs [17] and relays visual climbing fiber input relevant for vestibular-ocular reflex (VOR) tuning to the cerebellum via the inferior olive [9, 10]. A strategic lesion to AOS-related pretectal circuits deprives the cerebellum of visual-related inputs to match to vestibular signals—a nystagmogenic mechanism also proposed for oculopalatal tremor and developmental strabismus [4, 16].

The interplay between vision, translational VOR and vergence is paramount for depth perception during 3D head motion [4, 14]. Similar to a vestibular *roll* plane imbalance causing vertical skew deviation [4, 14], an imbalance in the translational *fore-aft* plane may cause esodeviation, which has been formalized as the “horizontal skew deviation” of developmental strabismus [16]. Skew deviations are associated with translational VOR deficits[14] and frequently accompany esodeviation and CRN [5, 6], like in our case. Finally, both oscillations’ striking gaze-position dependence suggests a vestibular coordinate framework, because vestibular nystagmus increases when gaze axis and culprit semicircular canal planes align [4]. This is mirrored by abnormal central vestibular findings in subjects with developmental strabismus [18].

Rooted in Occam’s razor, we argue that the observed parallels to the hallmarks of developmental strabismus—namely esodeviation with increase in upgaze, vertical divergence with inappropriate inferior oblique activation and fixation-dependent, disconjugate nystagmus—are not mere coincidence. Instead, we propose that this stereotypical pattern echoes a shared visuo-vestibular circuit mechanism with an essential hub region in the paracommissural midbrain. This fits with contemporary experimental results highlighting midbrain–cerebellar circuits as key drivers of developmental strabismus patterns [19]. Expanded circuit-level studies may further illuminate shared pathomechanisms and thereby potentially open new avenues for translational and therapeutic research.

### Limitations

Based on a single case report, generalizability is limited. We aimed to mitigate this by incorporating longitudinal assessments and employing granular phenotyping techniques facilitating comparisons to experimental studies. Due to technical constraints, vertical videooculographic recordings were only available in a binocularly averaged format, unable to fully capture the short-lived vertically disconjugate oscillations observed in the initial clinical presentation. However, we supply a high-resolution clinical video illustrating the clinical findings.

### Supplementary Information

Below is the link to the electronic supplementary material.

Supplementary file1 (DOCX 501 KB)

Supplementary file2 (PNG 4850 KB)

Supplementary file3 (MP4 81701 KB)

## Data Availability

Non-identifiable data are available upon reasonable request to the corresponding author. The MR-histological atlas is openly available at https://ftp.bigbrainproject.org/bigbrain-ftp/BigBrainRelease.2015/3D_Volumes/MNI-ICBM152_Space.

## Abbreviations

AOS: Accessory optic system
CRN: Convergence(-retraction) nystagmus
HSSN: Hemi-seesaw nystagmus
INC: Interstitial nucleus of Cajal
OTR: Ocular tilt reaction
PC: Posterior commissure
riMLF: Rostral interstitial nucleus of the medial longitudinal fasciculus
SSN: Seesaw nystagmus
TVOR: Translational vestibulo-ocular reflex
VOG: Videooculography
VOR: Vestibular-ocular reflex
