# Supplementary material for: Midbrain lesion-induced disconjugate gaze: a unifying circuit mechanism of ocular alignment?
Source: J Neurol. 2024 Feb 14;271(5):2844–9. doi: 10.1007/s00415-023-12155-6 (PMC11055718; doi:10.1007/s00415-023-12155-6)
Supplement: Supplementary file 1 — Supplementary file1 (DOCX 501 KB) [file 415_2023_12155_MOESM1_ESM.docx]

**Midbrain lesion-induced disconjugate gaze:**

**a unifying circuit mechanism of ocular misalignment?**

Friedrich, M.^1,2,3, †^, Schappe, L.^4,†^, Prasad, S.^5^, Friedrich, H.^1,2^, Fox, MD.^1,2^, Zwergal, A.^6,7^, Zee, DS.^8^, Faßbender, K.^4^, Dillmann, U.^4^

# Supplementary material


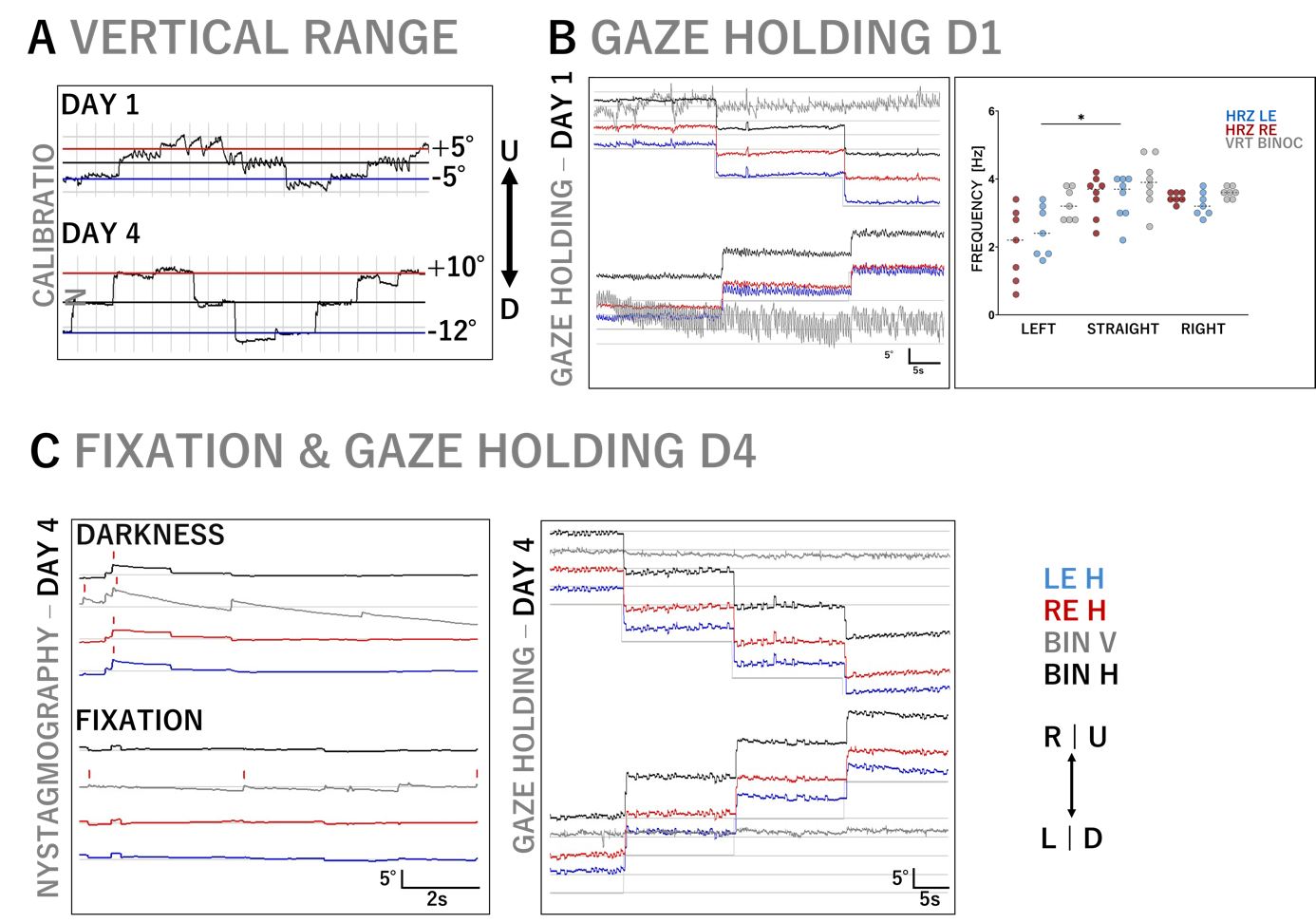


Supplementary Figure 1. Additional videooculographic findings. **A.** Upon calibration, vertical gaze limitation to ~5° in the acute phase is largely resolved at follow-up assessment (>10°). **B.** In the acute phase, vertical and horizontal oscillations are significantly attenuated in left gaze (p< .05). **C.** In the follow-up assessment on day 4, vertical nystagmus frequency is reduced to <1Hz in darkness and no nystagmus is detectable with fixation in light or gaze holding.

## Methods

### Videonystagmography

For quantitative eye movement analysis, a binocular infrared videooculography system operating at a spatial resolution of 0.1˚ (horizontal and vertical) and 60Hz sampling rate (Vestlab OS software, Hortmann Vestlab 100 hardware, VG-40 video glasses, Otometrics) was used as per standard clinical routine protocols. Briefly, the patient was seated in a quiet room with a projection-patient distance of 137 cm in front of a screen for stimulus presentation (projection height: 100 cm, projection width: 150 cm, Frame rate: 60/s). A screen stimulus based binocular calibration was conducted before the following paradigms were sequentially tested: nystagmus recording with and without fixation (i. e. in complete darkness), horizontal and vertical smooth pursuit and saccades, horizontal optokinetic nystagmus at 15, 30 and 45°/s.

### Statistical analysis

Mean frequencies and amplitudes were calculated based on two second data bins exported by proprietary VOG software (Vestlab OS). Graphpad Prism Version 9^1^ was used for statistical computations and illustrations.

### Neuroimaging

Axial and coronal diffusion-, T1- and T2-weighted brain MRI data acquired in the acute setting was used for lesion reconstruction and topographical analysis. Information from all sequences was utilized to manually trace and normalize the patient’s ischemic lesion into Montreal Neurological Institute (MNI152) space with respect to neighboring topographical structures (thalamus, red nucleus, aqueduct, third ventricle, periaqueductal grey, posterior commissure). Midbrain regions of interest such as the rostral interstitial nucleus of the medial longitudinal fasciculus (riMLF) and interstitial nucleus of Cajal (INC) have previously been segmented by an expert in brainstem anatomy (MF) as previously described^2^. The resulting final lesion mask and ROIs were reviewed by a junior and two senior neurologists (LS, KD, AZ). The segmentations were then overlaid with the Juelich BigBrain histological whole brain atlas at 400 micrometer resolution^3,4^, three-dimensionally reconstructed using FSLeyes^5^.

# Literature

1. GraphPad Software. GraphPad Prism.

2. Friedrich, M. U. *et al.* Current-dependent ocular tilt reaction in deep brain stimulation of the subthalamic nucleus: Evidence for an incerto-interstitial pathway? *Eur. J. Neurol.* (2022) doi:10.1111/ene.15257.

3. Human brain - Brain atlases. *Ebrains* https://www.ebrains.eu/brain-atlases/reference-atlases/human-brain/.

4. Amunts, K., Mohlberg, H., Bludau, S. & Zilles, K. Julich-Brain: A 3D probabilistic atlas of the human brain’s cytoarchitecture. *Science* **369**, 988–992 (2020).

5. Jenkinson, M., Beckmann, C. F., Behrens, T. E. J., Woolrich, M. W. & Smith, S. M. FSL. *NeuroImage* **62**, 782–790 (2012).
