# Supplementary figures and images for: Midbrain lesion-induced disconjugate gaze: a unifying circuit mechanism of ocular alignment?
Source: J Neurol. 2024 Feb 14;271(5):2844–9. doi: 10.1007/s00415-023-12155-6 (PMC11055718; doi:10.1007/s00415-023-12155-6)

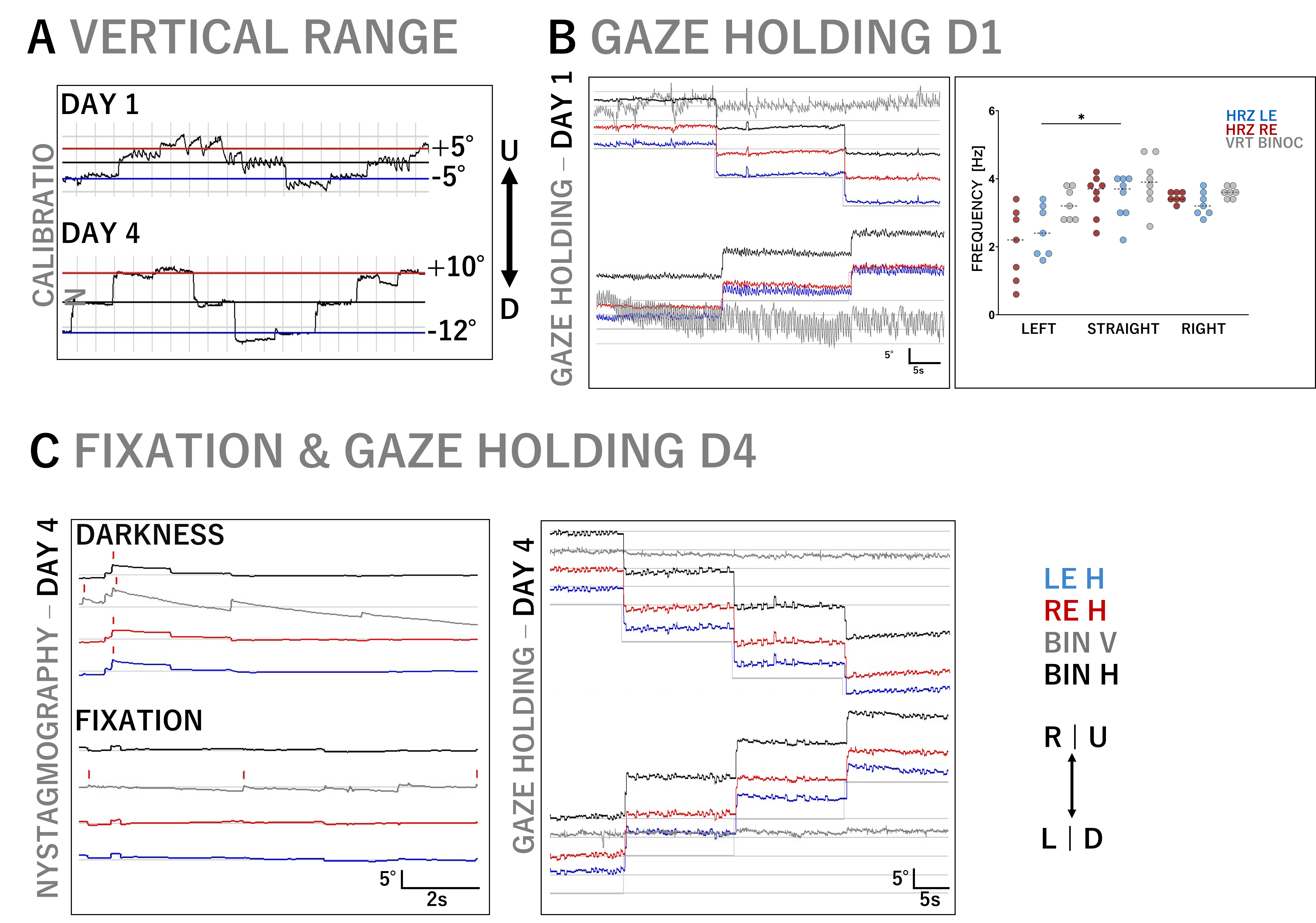

Supplement: Supplementary file 2 — Supplementary file2 (PNG 4850 KB) [file 415_2023_12155_MOESM2_ESM.png]
